# Combining EXAFS and Computer Simulations to Refine the Structural Description of Actinyls in Water

**DOI:** 10.3390/molecules25225250

**Published:** 2020-11-11

**Authors:** Sergio Pérez-Conesa, José M. Martínez, Rafael R. Pappalardo, Enrique Sánchez Marcos

**Affiliations:** Departamento de Química Física, Universidad de Sevilla, 41012 Sevilla, Spain; sperezconesa@gmail.com (S.P.-C.); josema@us.es (J.M.M.); rafapa@us.es (R.R.P.)

**Keywords:** actinyls in water, EXAFS, MD simulations, force field development, FEFF, electron correlation

## Abstract

EXAFS spectroscopy is one of the most used techniques to solve the structure of actinoid solutions. In this work a systematic analysis of the EXAFS spectra of four actinyl cations, [UO_2_]^2+^, [NpO_2_]^2+^, [NpO_2_]^+^ and [PuO_2_]^2+^ has been carried out by comparing experimental results with theoretical spectra. These were obtained by averaging individual contributions from snapshots taken from classical Molecular Dynamics simulations which employed a recently developed [AnO_2_]^2+/+^ –H_2_O force field based on the hydrated ion model using a quantum-mechanical (B3LYP) potential energy surface. Analysis of the complex EXAFS signal shows that both An-O${}_{\mathrm{yl}}$ and An-O${}_{W}$ single scattering paths as well as multiple scattering ones involving [AnO_2_]^+/2+^ molecular cation and first-shell water molecules are mixed up all together to produce a very complex signal. Simulated EXAFS from the B3LYP force field are in reasonable agreement for some of the cases studied, although the *k*= 6–8 Å${}^{-1}$ region is hard to be reproduced theoretically. Except uranyl, all studied actinyls are open-shell electron configurations, therefore it has been investigated how simulated EXAFS spectra are affected by minute changes of An-O bond distances produced by the inclusion of static and dynamic electron correlation in the quantum mechanical calculations. A [NpO_2_]^+^−H_2_O force field based on a NEVPT2 potential energy surface has been developed. The small structural changes incorporated by the electron correlation on the actinyl aqua ion geometry, typically smaller than 0.07 Å, leads to improve the simulated spectrum with respect to that obtained from the B3LYP force field. For the other open-shell actinyls, [NpO_2_]^2+^ and [PuO_2_]^2+^, a simplified strategy has been adopted to improve the simulated EXAFS spectrum. It is computed taking as reference structure the NEVPT2 optimized geometry and including the DW factors of their corresponding MD simulations employing the B3LYP force field. A better agreement between the experimental and the simulated EXAFS spectra is found, confirming the a priori guess that the inclusion of dynamic and static correlation refine the structural description of the open-shell actinyl aqua ions.

## 1. Introduction

Actinoid cations have a rich solution chemistry even in pure water which is very important technologically and environmentally [1,2]. Reprocessing of spent nuclear fuel involves several actinoids mainly under the form of actinyl aqua ions [AnO_2_(H_2_O)_m_]^q+^ [3,4,5,6,7,8,9,10]. In the actinyl motif, the actinoid cation, in oxidation state V or VI, bonds convalently to two oxygen atoms (“O${}_{\mathrm{yl}}$”) forming a linear unit. In aqueous solution, the equatorial plane perpendicular to this axis contains typically five water molecules coordinated to the metal atom. Knowledge of the chemistry of actinyl cations is key in the recovery of minor actinoids of high radiotoxicity from spent nuclear fuel [10].

X-ray absorption spectroscopy, particularly Extended X-ray absorption fine structure (EXAFS), is a powerful experimental technique to characterize the solvation around metal ions. The technique provides short range structural information around them in solution with remarkable accuracy and sensitivity, among them actinoids [11,12,13]. In particular, EXAFS has a structural precision in the hundredth of an angstrom in determining coordination distances around an absorbing atom. In addition, the technique is element specific and can handle submilimolar concentrations of the analyte. Unfortunately, the price to pay for its high precision is the difficulty in the fitting and interpretation of complex signals exhibiting multiple contributions, as those of actinyls. Analogies with lighter lanthanoids are often made [13] or theoretical tools are used giving rise to many interesting insights [11,14,15,16,17]. Conversely, the simulation of XAS spectra from molecular dynamics (MD) trajectories and their comparison to experiment has proven to be an useful tool to assess the accuracy of the intermolecular potentials employed in the simulation and to interpret experimental results [18,19,20,21].

In 2016 we presented a new DFT-level classical interaction potential for the [UO_2_]^2+^, pentahydrate [16] based on the Hydrated Ion model [11,22]. With the developed force field we carried out classical MD simulations in which we were able to reproduce a variety of experimental data [16]. Particularly, the model satisfactorily reproduces the experimental EXAFS spectrum of uranyl in water. We decomposed the spectrum in two main single scattering path components: an intense and slowly decaying signal corresponding to U-O${}_{\mathrm{yl}}$ paths, and another less intense, higher frequency and fast-decaying signal corresponding to U-O${}_{W}$ paths. Multiple scattering paths involving crossed U-O${}_{\mathrm{yl}}$ and U-O${}_{W}$ paths were found to be minor contributions. The methodology to build intermolecular potentials was extended to other actinyl cations –[NpO_2_]^2+^, [NpO_2_]^+^, [PuO_2_]^2+^ and [AmO_2_]^2+^– providing a general view of actinyl hydration and the effect of changing properties when going from the dication to the monocation [23]. Using the americyl hydrated ion model we studied the nature of the first Am(VI)/Am(III) mixture spectrum recorded [24] by Riddle et al. [25]. Despite the importance of XAS spectroscopy in actinyl chemistry, to the best of our knowledge there are only MD simulated spectra devoted to the case of uranyl in water and in organic phase [16,26,27,28]. The only exception are the XANES spectra calculated for neptunyl by den Auwer et al. [29] with a rigid aqua ion structural model.

**Figure 1:**
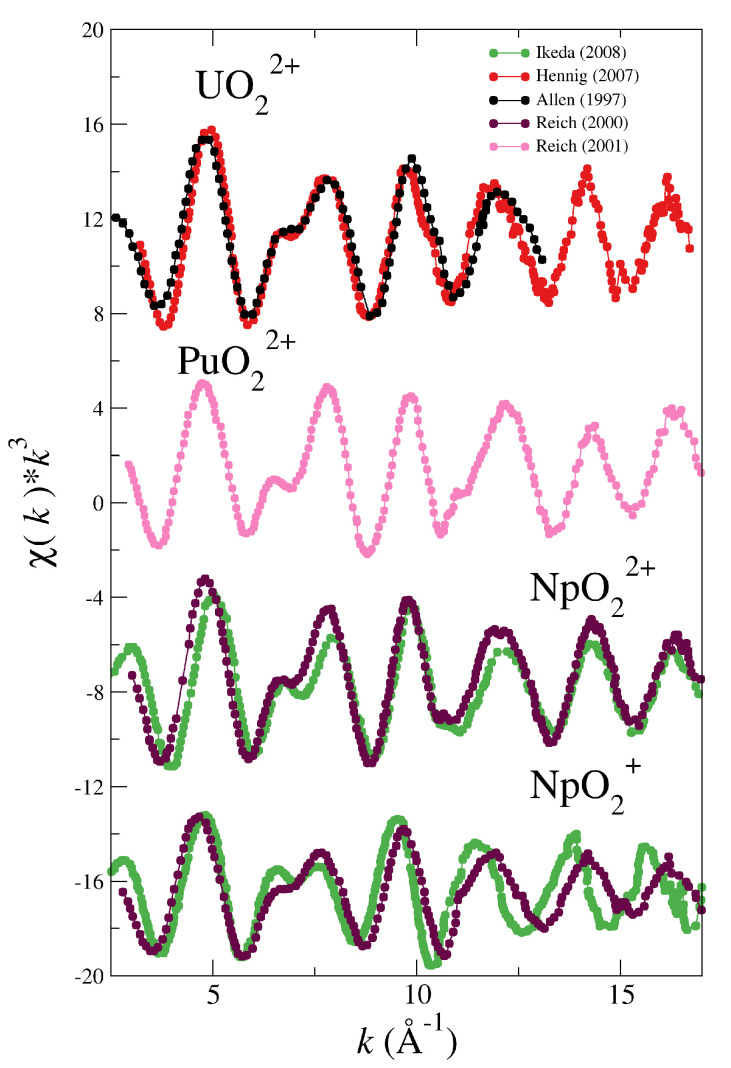
Experimental $k^{3}$-weighted EXAFS spectra recorded for aqueous solutions of [UO_2_]^2+^ (Allen (1997) [5] (black dotted line) and Henning (2007) [30] (red dotted line)), [PuO_2_]^2+^ (Reich (2001) [33] (pink dotted line)), [NpO_2_]^2+^ and [NpO_2_]^+^ (Ikeda (2008) [32] (green dotted line) and Reich (2000) [31] (magenta dotted line))

This work aims the systematic study of MD-based simulated EXAFS spectra obtained by means of the application of our actinyl force fields in solution for the series of actinyls cations, [UO_2_]^2+^, [NpO_2_]^2+^, [NpO_2_]^+^ and [PuO_2_]^2+^. Figure 1 shows the experimental $k^{3}$-weighted EXAFS spectra of the actinyls recorded by different groups during the last twenty years in independent experiments. All experimental EXAFS spectra were obtained at highly acidic pH, with non-coordinating counterions and an actinyl concentration of ∼50–10 mM [5,30,31,32,33]. For the actinyls studied there are two spectra recorded by different groups, except for plutonyl where only one spectrum is available in the literature. The comparison between spectra is quite similar for uranyl [5,30] and neptunyl(VI) [31,32], but in the neptunyl(V) case a larger discrepancy is found [31,32]. It must be pointed out that the pair of neptunyl spectra for each oxidation state were recorded by the same two groups in the same experiments [31,32]. The larger discrepancy in the case of neptunyl(V) shows the difficulty of this kind of measurements. In both cases, dilute aqueous solutions were measured and no complexation or other medium effects could be responsible of this discrepancy, but rather the intrinsic difficulty to carry out measurements of this type of highly-radioactive samples.

All the actinyls of this study have open-shell electron configurations except uranyl. We will evaluate the goodness of the DFT mean field treatment of static electronic correlation effects to provide an accurate enough EXAFS spectrum prediction for open-shell actinyls, since DFT has shown the ability of our model to reproduce many other experimental properties [23,34,35]. To get insight into this question and check the EXAFS sensitivity to minute structural changes, multi-reference NEVPT2 [36,37,38] computations have been performed to build a [NpO_2_]^+^−H_2_O interaction potential based on this quantum-mechanical level.

## 2. Computational Methods

### 2.1. Quantum Chemical Calculations

The interaction potentials published in our previous article [23] were parameterized at a DFT level of theory: B3LYP [39,40]/aug-cc-PVDZ [41] with Stuttgart relativistic effective core pseudopotentials [42] using Gaussian09 [43]. The B3LYP functional has given reasonable interaction energies and molecular geometries for actinyls [44,45,46] both open and closed shells [47,48]. In our previous work we showed that an interaction potential parameterized at that level of theory was able to reproduce satisfactorily different experimental properties.

To get insight into the impact that static and dynamical electron correlation may have on the geometrical structure and dynamical and structural disorder of the close environment of the actinyls, NEVPT2 calculations, which incorporate both dynamical and static electronic correlation were carried out using ORCA [49] program. Although these calculations are too expensive to envisage the full force field development for every case, we shall use them to check the influence of electron correlation in the actinyl geometry and as a result in their simulated EXAFS spectra. The active space chosen was the set of atomic-like f-orbitals in addition to the molecular orbitals resulting from combining actinide f-orbitals and O${}_{\mathrm{yl}}$ p-orbitals. This resulted in CASSCF(7,10) configurations for NpO_2_^2+^ and CASSCF(8,10) configurations for NpO_2_^+^ and PuO_2_^2+^. Since the ground states are degenerate, calculations were carried out using a state average over the degenerate states excluding excited states. The perturbational step of the calculation was done using quasi-degenerate perturbation theory. The basis sets used were ma-def2-TZVP for O, def2-SVP for H and SD(60,MWB)//DEF-TZVP for actinoids [50,51,52]. The calculations were accelerated using the RI and RIJK pseudospectral methods with “autoaux” auxiliary basis sets. Geometry optimizations due to the lack of analytical gradients were performed numerically by evenly changing the M-O${}_{\mathrm{yl}}$ and M-O${}_{W}$ distances in a 2D grid with a step of ∼0.005Å.

### 2.2. Molecular Dynamics Simulations

MD simulations were run in a similar way to our previous studies on actinyls [16,23,24]. A single pentahydrated actinyl ion, [AnO_2_(H_2_O)_5_]^2^+, (An=U(VI),Np(VI),Np(V),Pu(VI)) and 1495 TIP4P water molecules were placed in a cubic box at the experimental water density. The simulations were run at 300 K and 1 atm in the NPT ensemble using the Noosé–Hoover thermostat and barostat both of them with τ=0.5ps. Non-bonded interactions were cut at 14 Å and electrostatic interactions were computed using the Ewald summation. The equations of motion were integrated using a 1 fs timestep for a total time of 5 ns. All simulations were run using DL_POLY Classic [53]. The convergence of MD trajectories has been previously checked by the analysis of structural, energetic and dynamic properties of the actinyl cations as shown in Refs. [16,23].

The first set of interaction potentials used in this work were developed previously [16,23,24]. They are based on the hydrated ion model proposed by our group over 20 years ago [22]. In this model we consider the ion and its first hydration shell as the solute rather than the naked ion. In this way first-shell water molecules have different atom types and interaction potentials than bulk water molecules. This allows the first-shell molecules to have different partial charges, i.e., incorporating charge transfer from the metal, and different non-bonded interactions with the metal and bulk water molecules. Then we are able to incorporate charge transfer and polarization effects into an effective non-polarizable site–site interaction potential. The caveat to this model is that if a first-shell water molecule were to leave the first shell it would render the system unphysical. Nevertheless, for many ions, as it is the case for actinyls, the characteristic time for this phenomenon is much longer than the simulation time, i.e., first-shell mean residence times well longer than the simulation time.

The interaction potentials were all parameterized from B3LYP quantum chemical calculations. The interaction within the molecular ion (IMC, intramolecular cation) and the interaction of the molecular ion with its first shell (IW1, ion first-shell water) were parameterized specifically for each ion. The interaction of the hydrated ion and bulk water molecules (HIW, hydrated ion water) was parameterized only for the uranyl case. For the rest of the actinoids the non-electrostatic component of the HIW of uranyl was used since it has proven to be fairly similar across the studied actinoids [23]. Non-electrostatic water-water interactions within the first shell were modeled in the same way as in the TIP4P model. Finally, the TIP4P water model was used to model bulk water molecule interactions. Therefore the energy of the system is defined by the following expression:

(1) $$E=E_{\mathrm{IMC}}+E_{\mathrm{IW}1}+E_{W_{I}-W_{I}}+E_{\mathrm{HIW}}+E_{\mathrm{TIP}4P}$$

The parameterized terms of the potential were given the functional form of $r^{-n}$ with n=4,6,8,12 plus the coulombic term. As an example we present below the functional form of the IW1 potential:

(2) $$E_{\mathrm{IW}1}=\sum_{i}^{\begin{array}{c} {\mathrm{AnO}}_{2}\;\\ \mathrm{sites} \end{array}}\frac{C_{4}^{\mathrm{iO}}}{r_{\mathrm{iO}}^{4}}+\frac{C_{6}^{\mathrm{iO}}}{r_{\mathrm{iO}}^{6}}+\frac{C_{8}^{\mathrm{iO}}}{r_{\mathrm{iO}}^{8}}+\frac{C_{12}^{\mathrm{iO}}}{r_{\mathrm{iO}}^{12}}+\sum_{i}^{\begin{array}{c} {\mathrm{AnO}}_{2}\;\\ \mathrm{sites} \end{array}}\;\sum_{j}^{\begin{array}{c} \mathrm{Water}\;\\ \mathrm{sites} \end{array}}\frac{q_{i}q_{j}}{r_{\mathrm{ij}}}$$

Due to the paramagnetic nature of the actinyls, except the uranyl case, in this work we have explored how the inclusion of static and dynamic electron correlation in the wavefunction used to fit the force field may affect to the solution properties and the shape of the simulated EXAFS spectra. To fulfill this task we have built a second NpO_2_^+^ interaction potential using the quantum-mechanical energies derived from NEVPT2 calculations rather than B3LYP ones. All the parameters of the interaction potentials including the new neptunyl(V) full NEVPT2 potential are collected in the Supporting Information (SI).

### 2.3. Simulated XAS Spectra

Molecular dynamics simulations are a useful tool to help in the interpretation and analysis of complex EXAFS spectra [15,21,54]. Conversely, comparison of experimental spectra with simulated ones generated from an ensemble of MD configurations is a useful and sensitive tool to validate force fields [18,21]. 200 evenly-spaced configurations of [AnO_2_]^2+/+^ were extracted from the MD trajectories, i.e., the time interval between two consecutive snapshots is 25 ps that guarantees non-correlated statistical information. Previous studies have shown that selecting a higher number of snapshots, e.g., 500 or 1000 snapshots, produces the same EXAFS simulated spectrum [16,24]. To illustrate this convergence in one of the studied cases, Figure S3 of SI shows the B3LYP simulated spectra by averaging 200 and 500 snapshots for the plutonyl case. The configurations included water molecules up to the first solvation shell since we have found the second shell to have no-influence in the spectra. Average $L_{\mathrm{III}}$-edge spectra were obtained from the individual spectra using the FEFF 9.6 code [55] including multiple scattering up to four-legged paths. Details of the spectrum simulation method can be found elsewhere [11,21,24]. In addition, an example of the FEFF input files can be found in the SI. A value of $S_{0}^{2}$ = 0.81 has been assumed for the simulated spectra, and $\Delta E_{0}$ of −7 or −8 eV have been applied in order to match the first resonance of the experimental spectrum.

## 3. Results and Discussion

**Figure 2:**
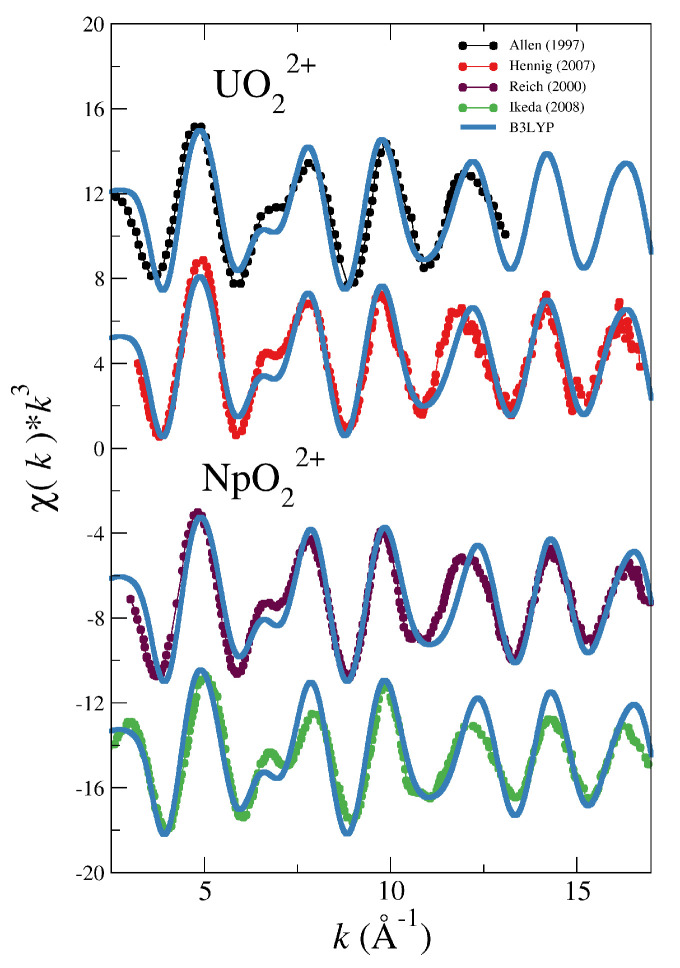
Simulated (blue solid line) and experimental (dots) L${}_{\mathrm{III}}$-edge k${}^{3}$-weighted EXAFS spectra for UO_2_^2+^ and NpO_2_^2+^ in water. The experimental EXAFS are taken from Allen (1997) [5] (black dotted line) and Henning (2007) [30] (red dotted line) for uranyl, and from Reich (2000) [31] (magenta dotted line) and Ikeda (2008) [32] (green dotted line).

**Figure 3:**
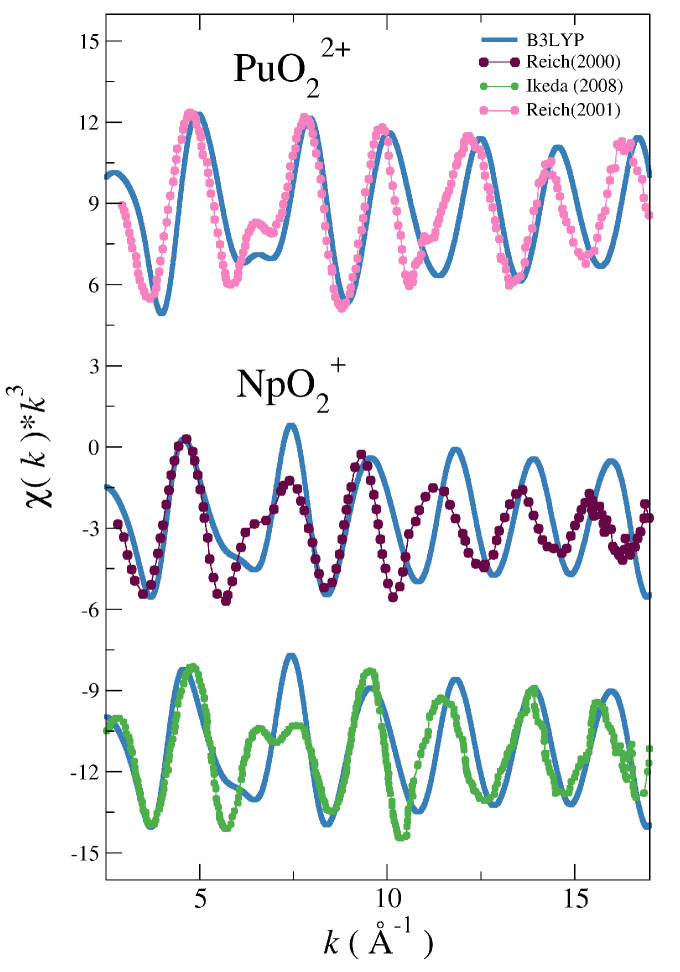
Simulated (blue solid line) and experimental (dots) L${}_{\mathrm{III}}$-edge k${}^{3}$-weighted EXAFS spectra for PuO_2_^2+^ and NpO_2_^+^ in water. The experimental EXAFS are taken from Reich (2001) [33] (pink dotted line) for plutonyl, and from Reich (2000) [31] (magenta dotted line) and Ikeda (2008) [32] (green dotted line).

Figure 2 and Figure 3 show the comparison between the experimental spectra of the different actinyls [5,30,31,32,33] and the simulated spectra obtained with the force field derived from B3LYP potential energy surfaces (blue lines) developed in our previous works on actinyls in water [16,23].

**Figure 4:**
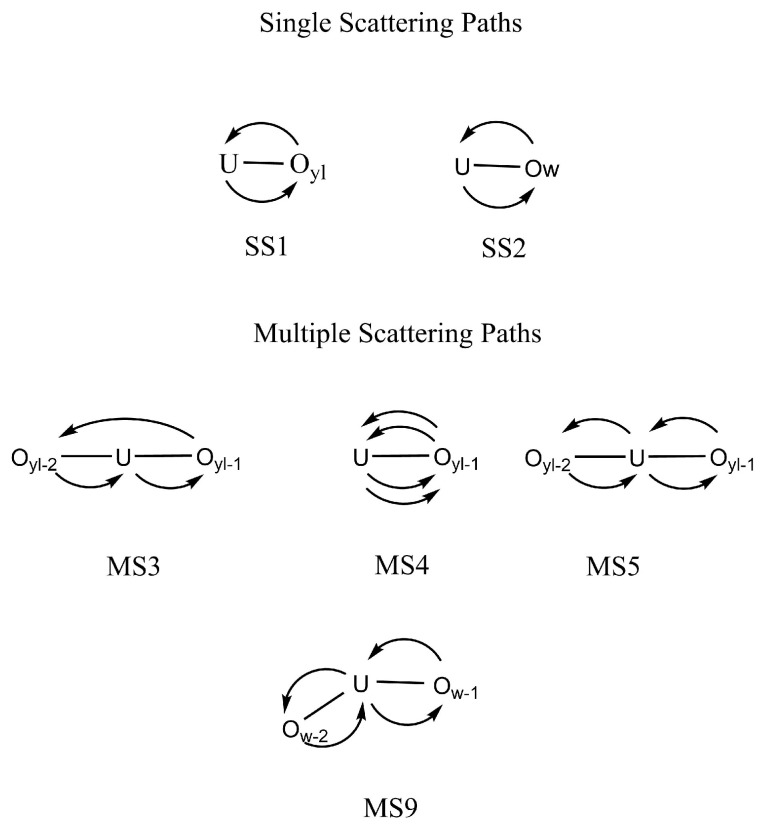
Main single scattering (SS) and multiple scattering (MS) paths contributing to the EXAFS signal.

The cases of the UO_2_^2+^ and NpO_2_^2+^ collected in Figure 2 show a good general agreement with experiment for uranyl and a reasonable agreement for neptunyl(VI) bearing in mind that there are two different experimental spectra to compare for each actinyl. When considering the comparison for the PuO_2_^2+^ and NpO_2_^+^ cases, shown in Figure 3, deviations from experimental spectra are significant, in particular in the case of the neptunyl(V). Actinyls in aqueous solution have complex EXAFS spectra due to the superposition of several scattering paths with rather different patterns as shown in Figure 4. This set of paths contributes significantly to the EXAFS signal.

**Figure 5:**
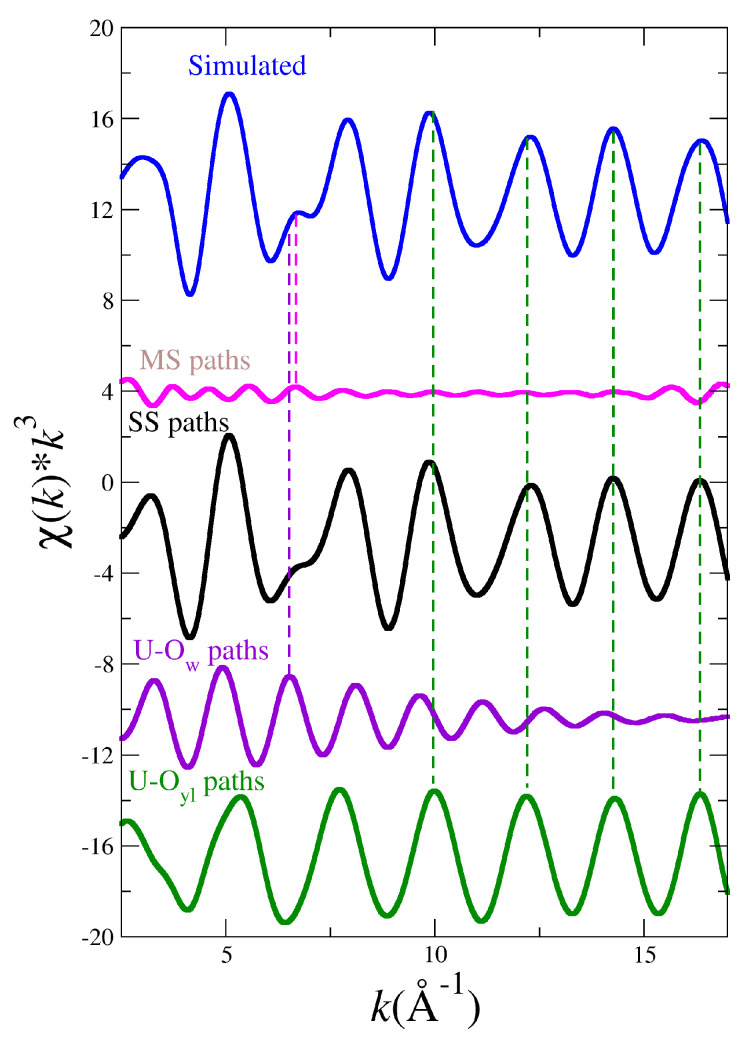
Simulated L${}_{\mathrm{III}}$-edge k${}^{2}$-weighted EXAFS spectrum for U(VI) (blue line) and its decomposition in MS scattering (pink line) and SS scattering (black line) contributions, as well as the U-O${}_{w}$ (magenta line) and U-O${}_{\mathrm{yl}}$ (green line) SS contributions.

Figure 5 shows the simulated EXAFS spectrum corresponding to the uranyl (blue line) and its decomposition into the single scattering (SS, black line) and multiple scattering (MS, pink line) contributions. The SS contribution comes from the superposition of two SS U-O${}_{\mathrm{yl}}$ (green line) and U-O${}_{w}$ (magenta line) paths. The MS contribution is due to the set of relevant MS paths collected in Figure 4. It is observed how the hump and its surrounding region, 6–8 Å${}^{-1}$, is mainly the result of the SS U-O${}_{w}$ and MS paths (see magenta and pink vertical lines), whereas the high *k*-region is dominated by the SS U-O${}_{\mathrm{yl}}$ paths (see green vertical lines).

This type of decomposition is representative of the actinyl series. Two SS signals are ascribed to a slowly decaying An-O${}_{\mathrm{yl}}$ contribution with high intensity (see green line in Figure 5) and a higher frequency fast decaying An-O${}_{w}$ signal (see magenta line in Figure 5). In addition, a set of MS paths (see pink line in Figure 5) associated to the AnO_2_^2+^ cation and a path involving the first-shell water molecules contribute to make an involved spectrum [16,56]. Destructive interference of the main components generates the characteristic shoulder around *k* = 6.5 Å^−1^, whereas at high *k* values the spectrum is dominated by the An-O signal (see magenta line in Figure 5). In addition, a set of MS paths (see pink line in Figure 5) associated to the AnO_2_^2+^ cation and a path involving the first-shell water molecules contribute to make an involved spectrum [16,56]. Destructive interference of the main components generates the characteristic shoulder around *k* = 6.5 Å^−1^, whereas at high *k* values the spectrum is dominated by the An-O${}_{\mathrm{yl}}$ component to a large extent.

Once analyzed the main structural factors responsible of the EXAFS spectrum, since the differences between simulated and experimental spectra became larger as the series progresses and the spin multiplicity of the complex increases, it became apparent that a multireference PES including static and dynamic correlation could help to solve the problem. We hypothesized that the B3LYP An-O${}_{\mathrm{yl}}$ distances were systematically too short and, probably as a consequence of the larger congestion around the metal center, the An-O${}_{W}$ B3LYP distances were too long. The oxo-bond is markedly covalent in character, as a consequence an underestimation of its static electronic correlation causes a shortening of the An-O${}_{\mathrm{yl}}$.

### 3.1. Simulated EXAFS Spectrum Based on A NEVPT2 Force Field for NpO_2_^+^ in Aqueous Solution

**Figure 6:**
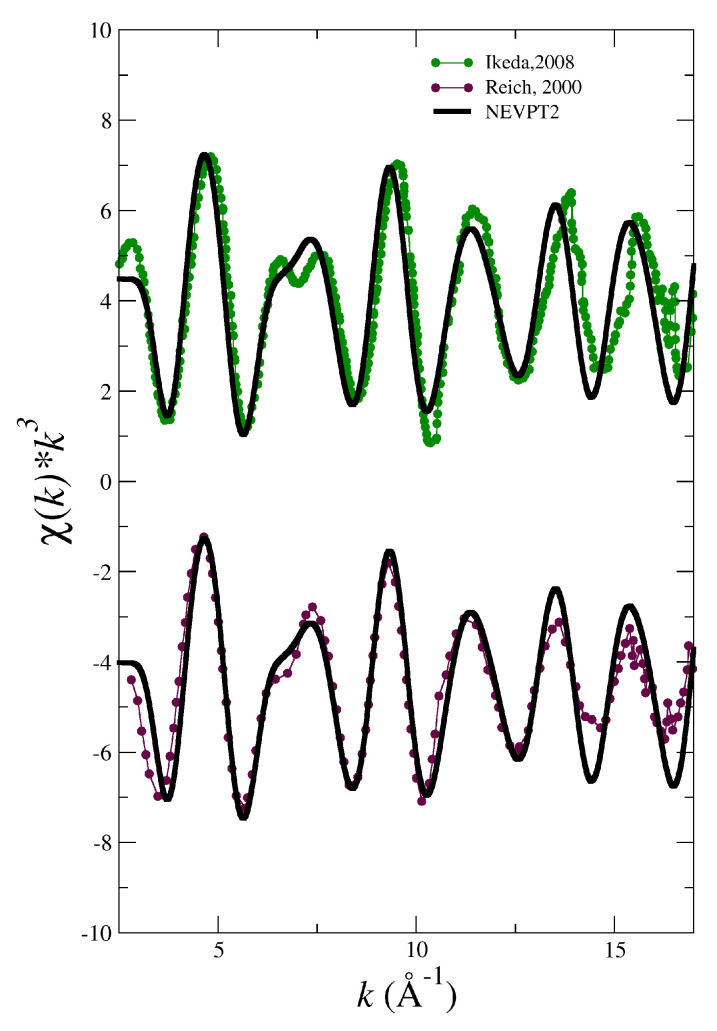
Simulated (black solid line) vs experimental [31,32] (dots) L${}_{\mathrm{III}}$-edge k${}^{3}$-weighted EXAFS spectra for NpO_2_^+^. The simulated spectra is obtained using the NEVPT2-level interaction potential developed for the monovalent neptunyl cation in water.

To check the hypothesis that a better agreement of the simulated NpO_2_^+^, NpO_2_^2+^ and PuO_2_^2+^ spectra with the experimental ones could be achieved dealing with a multireference representation of their wavefunctions, a classical interaction potential for NpO_2_^+^−H_2_O based on a NEVPT2 PES has been developed. The spectrum derived from the set of MD-simulation snapshots using the NEVPT2 force field is presented in Figure 6 (black solid line). It is clearly superior to the B3LYP-level counterpart (see Figure 3), in particular the simulated spectrum is in good agreement with the experimental one of Reich et al. [31] except for the high intensity of the signal at high *k*-value (k≥ 12.5 Å${}^{-1}$).

Table 1 collects the optimized An-O${}_{\mathrm{yl}}$ and An-O${}_{W}$ distances obtained at the QM level (B3LYP-opt, MP2-opt/NEVPT2-opt) for the isolated actinyl aqua ion, as well as the values derived from the use of the developed force field (B3LYPPOT-opt, NEVPOT-opt) and the most likely distances and their corresponding Debye–Waller factors derived from the MD simulations (B3LYP-MD, NEVPT2-MD). The geometry of the [NpO_2_(H_2_O)_5_]^+^ at the B3LYP-level has Np-O${}_{\mathrm{yl}}$ and Np-O${}_{W}$ distances of 1.78 Å and 2.59 Å respectively. As anticipated the NEVPT2-level optimized geometry has longer Np-O${}_{\mathrm{yl}}$ bonds ( 1.83 Å) and shorter Np-O${}_{W}$ distances (2.52 Å), this is also reflected in the distances obtained in the MD simulations: Np–O${}_{\mathrm{yl}}$ is 1.79 Å (B3LYP-MD) and 1.84 Å (NEVPT2-MD), whereas Np–O${}_{w}$ is 2.61 Å (B3LYP-MD) and 2.54 Å (NEVPT2-MD). We conclude for neptunyl(V) that the problem of our initial spectrum was that the reference DFT hydrated ion minimum energy geometry was not accurate enough for a very precise spectrum reproduction. EXAFS spectrum is so sensitive to structure that changes in a few hundreths of angstrom imply noticeable modifications in the EXAFS spectrum shape. A multireference QM method including both static and dynamic electron correlation is therefore necessary if a high accuracy is needed.

The change in distances of [NpO_2_(H_2_O)_5_]^+^ when going from B3LYP to NEVPT2 is also found in the cases of the other open-shell actinyl aqua ions, although this effect is less pronounced (cf. NpO_2_^2+^ and PuO_2_^2+^ data in Table 1).

### 3.2. Simulated EXAFS Spectrum Based on NEVPT2 Optimized Geometries

The cost of developing a force field at the NEVPT2 level prevents the generalization of the procedure adopted for NpO_2_^+^ to the other two open-shell cations, NpO_2_^2+^ and PuO_2_^2+^. However, the gained knowledge with NpO_2_^+^ led us to explore a less computationally demanding strategy to simulate the EXAFS spectra of these two actinyls in water. Then, we have combined the refined structural description given by the quantum-mechanical NEVPT2 level and the statistical average provided by the MD simulations. The procedure has been the computation of the EXAFS spectrum taking as reference the NEVPT2 optimized structure of the pentahydrate of the actinyl, considering the DW factors of the most representative SS and MS paths, displayed in Figure 4, derived from their corresponding B3LYP MD simulations, whose values are collected in Table 2.

**Figure 7:**
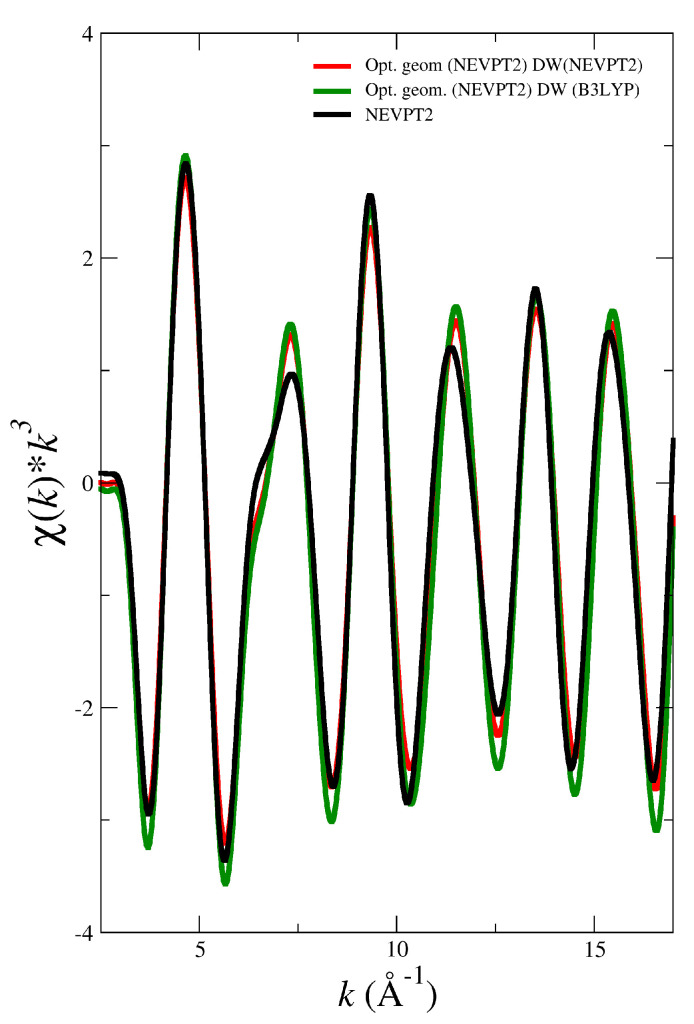
L${}_{\mathrm{III}}$-edge k${}^{3}$-weighted simulated EXAFS spectra for NpO_2_^+^ from MD simulation which uses the NEVPT2 force field (black line) and the NEVPT2 optimized geometry using the DW factors of the NEVPT2 MD simulation (red line) or the B3LYP MD simulation (green line).

To test this simplified procedure, first of all we have computed the EXAFS spectrum of NpO_2_^+^ in water using the optimized geometry for [NpO_2_(H_2_O)_5_]^+^ obtained by NEVPT2 and including the Debye-Waller factors of the most important paths derived either from the MD simulation using the NEVPT2 force field or the one using the B3LYP force field collected in Table 2. Figure 7 displays these two spectra together with the EXAFS spectrum obtained from the MD simulation using the NEVPT2 method. Interestingly, the agreement is quite satisfactory, something that can be understood when inspecting the DW factors of the B3LYP and NEVPT2 MD simulations for NpO_2_^+^ (see Table 2). Another conclusion can be drawn from Figure 7 is that solvent effects do not cause significant geometrical changes in the actinyl pentahydrates. This derives from the analysis of the EXAFS spectra obtained from the NEVPT2 MD simulation (black line) and that obtained from the NEVPT2 optimized geometry (red line) which formally represents gas phase geometry.

The good results found for NpO_2_^+^ led us to a further step applying this strategy to the NpO_2_^2+^ and PuO_2_^2+^ cases. We have not developed NEVPT2 force fields for these two actinyls in water, but the NEVPT2 optimized geometries of their pentahydrates have been located (see Table 1). Then, we have computed the EXAFS spectra for neptunyl(VI) and plutonyl(VI) taken as reference the NEVPT2 optimized geometries and using the DW values obtained in the B3LYP MD simulation for the set of SS and MS paths collected in Figure 4.

**Figure 8:**
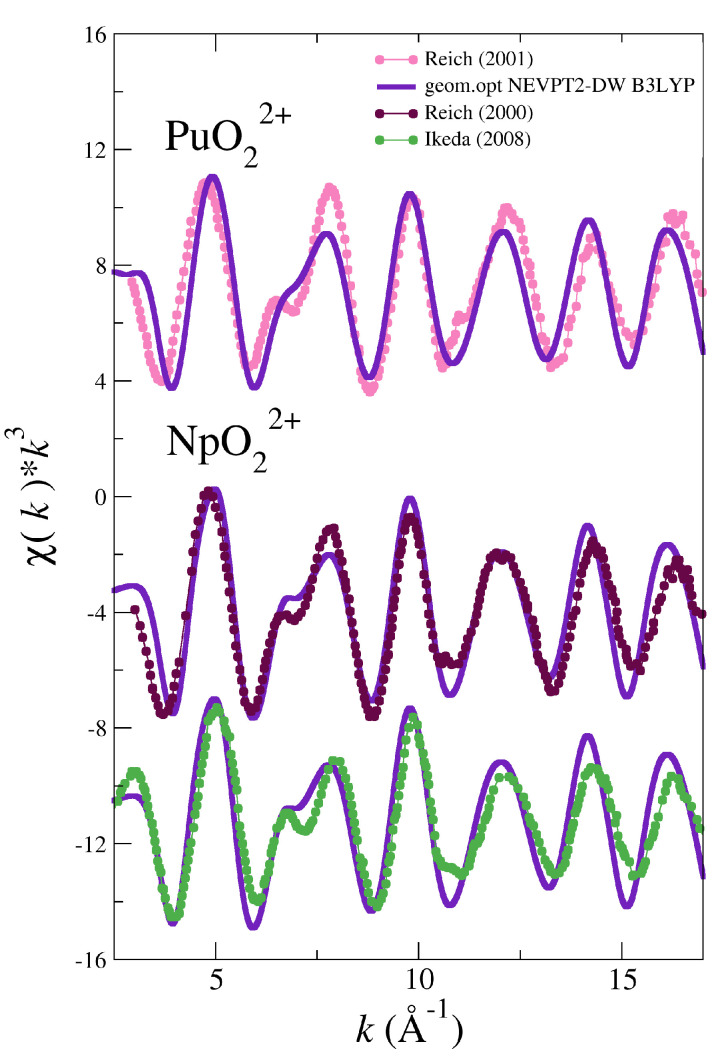
L${}_{\mathrm{III}}$-edge k${}^{3}$-weighted experimental and simulated EXAFS spectra for PuO_2_^2+^ and NpO_2_^2+^ from the NEVPT2 optimized geometries for their pentahydrates and using the DW factors of their corresponding MD simulations using the B3LYP force fields.

The simulated spectra are compared with the available experimental ones for these two actinyls in Figure 8. It must be noted how the positions of the different oscillations are now almost matching those of the experimental spectra. The simulated intensities have improved with respect to the B3LYP simulated spectra (cf. Figure 2 and Figure 3), although the intensities at high-*k* are overestimated with respect to the experimental signal. The challenging 6–8 Å${}^{-1}$ region of the double peak is now reasonably described, better in the NpO_2_^2+^ than in the PuO_2_^2+^ case. It is worth noting the case of UO_2_^2+^, for which the optimized geometry at the MP2 level has also been located. For a closed-shell molecule this computational level is equivalent to that employed for the predecent open-shell actinyls studied. The simulated spectrum using the MP2 geometry and the DW factors taken from the corresponding B3LYP MD simulation is very similar to the experimental spectra as shown in Figure S4 of SI, as well as to the corresponding spectrum derived from the B3LYP MD simulation (see Figure 2). The similarity, not found in the cases of the open-shell actinyls, can be understood by inspecting the distances for the [UO_2_(H_2_O)_5_]^2+^ in Table 1. *R*(AnO${}_{\mathrm{yl}})$ and *R*(AnO${}_{w}$) for B3LYP-MD and MP2-opt are much closer than in the cases of the open-shell actinyls, distances differ only by 0.01 Å and 0.02 Å, respectively.

In Table 1, we have included the EXAFS parameters obtained in this study and their experimental counterparts. It is clear that our theoretical parameters are significantly improved when the quantum-mechanical NEVPT2 method is used either to build a force field that is employed in the MD simulation, NpO_2_^+^ case, or optimized geometries at this level for NpO_2_^2+^ and PuO_2_^+^ are employed. This improvement is particularly strong for the An-O${}_{\mathrm{yl}}$ and An-O${}_{W}$ distances which are the dominant contributions to the spectra.

## 4. Conclusions

Regardless of the overall well behavior of our previously developed actinyl interaction potentials, based on B3LYP potential energy surfaces, with most experimental properties [16,23,24], their simulated EXAFS spectra do not reproduce adequately the experimental ones in all cases.

This work has shown the improvement in the theoretical computation of the EXAFS spectrum corresponding to the open-shell actinyls when instead of the B3LYP-based interaction potential, the NEVPT2-based interaction potential is used in the classical MD simulations, from which the structural information to simulate the EXAFS spectrum is taken. Interestingly, we have shown that a simplified procedure to avoid the specific building of a NEVPT2-based interaction potential, using the NEVPT2 optimized geometries and the DW factors taken from B3LYP MD simulation, gives satisfactory results. This has been proven for the case of the three open-shell actinyls considered in this study. The first-principles nature of the developed potential opens the door to build force fields based on calculations at higher quantum-mechanical levels. They might refine the structural description of the actinyl hydrates, as previously done in gas-phase studies of actinyls without water molecules that have examined the inclusion of scalar relativistic effects, spin-orbit coupling terms and coupled-cluster methods. [57,58,59]

From the structural analysis we obtain that the An-O${}_{\mathrm{yl}}$ distance in solution for the divalent uranyl, neptunyl and plutonyl is framed in the interval 1.76–1.77 Å, whereas for the monovalent neptunyl is 1.83–1.84 Å. The An-O${}_{w}$ distance interval for the divalent neptunyl and plutonyl, is 2.42–2.43 Å, a longer distance is observed in the uranyl case, 2.49–2.50 Å, and an even longer value for the monovalent neptunyl, 2.52–2.54 Å.

In conclusion, we found out that for actinyl aqua ions the structural precision of EXAFS is so high that in order to properly simulate their EXAFS spectrum the impact of static correlation effects on the open-shell actinyl structure must be taken into account.

## Figures and Tables

**Table 1:** Gas phase QM and Force Field optimizations and MD first-shell distances, and Debye-Waller factors ($\sigma^{2}$) for the studied actinyl aquaions. All experimental EXAFS spectra are obtained at highly acidic pH, with non-coordinating counterions and an actinyl concentration of ∼50 mM. The MD values are obtained from the RDF-maxima and variance. (distances in Å and DW in ${Å}^{2}$).

System		$r_{{\mathrm{AnO}}_{\mathrm{yl}}}$	$\sigma_{{\mathrm{AnO}}_{\mathrm{yl}}}^{2}$	$r_{{\mathrm{AnO}}_{w}}$	$\sigma_{{\mathrm{AnO}}_{w}}^{2}$
[UO_2_]^2^+(aq)
		Quantum Mechanics			
	B3LYP-opt	1.74		2.49	
	MP2-opt	1.77		2.50	
		Force Field			
	B3LYPPOT-opt	1.76		2.49	
	B3LYP-MD	1.76	0.0004	2.48	0.007
		Experimental			
	Hennig 2007 [30]	1.76 ± 0.02	0.002	2.41 ± 0.02	0.007
	Allen 1997 [5]	1.76 ± 0.01	0.002	2.41 ± 0.01	0.007
[NpO_2_]^2^+(aq)
		Quantum Mechanics			
	B3LYP-opt	1.73		2.48	
	NEVPT2-opt	1.77		2.42	
		Force Field			
	B3LYPPOT-opt	1.74		2.47	
	B3LYP-MD	1.74	0.0004	2.46	0.007
		Experimental			
	Ikeda 2008 [32]	1.76 ± 0.01	0.002	2.42 ± 0.01	0.006
	Reich 2000 [31]	1.754 ± 0.003	0.002	2.414 ± 0.006	0.006
[NpO_2_]^+^(aq)
		Quantum Mechanics			
	B3LYP-opt	1.78		2.59	
	NEVPT2-opt	1.83		2.52	
		Force Field			
	B3LYPPOT-opt	1.78		2.59	
	NEVPOT-opt	1.83		2.52	
	B3LYP-MD	1.79	0.0007	2.61	0.012
	NEVPT2-MD	1.84	0.0007	2.54	0.011
		Experimental			
	Ikeda 2008 [32]	1.84 ± 0.01	0.002	2.49 ± 0.01	0.007
	Reich 2000 [31]	1.822 ± 0.003	0.002	2.488 ± 0.009	0.006
[PuO_2_]^2^+(aq)
		Quantum Mechanics			
	B3LYP-opt	1.71		2.46	
	NEVPT2-opt	1.76		2.43	
		Force Field			
	B3LYPPOT-opt	1.71		2.47	
	B3LYP-MD	1.71	0.0006	2.45	0.009
		Experimental			
	Reich 2001 [33]	1.74 ± 0.01	0.001	2.42 ± 0.01	0.005

**Table 2:** DW factors of the main SS and MS paths of [AnO_2_(H_2_O)_5_]^+/2+^ in water derived from MD simulations (path description can be found in Figure 4). DW in ${Å}^{2}$.

		$\sigma^{2}$ (AnO${}_{2}$) × 10${}^{3}$ (MD Simulation)				
Path Index	Path Type	NpO_2_^+^ (B3LYP)	NpO_2_^+^ (NEVPT2)	NpO_2_^2+^ (B3LYP)	UO_2_^2+^ (B3LYP)	PuO_2_^2+^ (B3LYP)
1	O${}_{\mathrm{yl}}$-An	0.70	0.73	0.39	0.59	0.41
2	O${}_{w}$-An	10.8	10.5	6.29	9.05	7.15
3	O${}_{\mathrm{yl}-1}$-O${}_{\mathrm{yl}-2}$-An	1.31	1.57	0.74	1.36	0.89
4	O${}_{\mathrm{yl}-1}$-An-O${}_{\mathrm{yl}-1}$-An	2.79	2.91	1.54	2.35	1.64
5	O${}_{\mathrm{yl}-1}$-An-O${}_{\mathrm{yl}-2}$-An	1.33	1.54	0.74	1.35	0.86
9	O${}_{w-1}$-An-O${}_{w-2}$-An	15.8	14.9	9.53	12.1	9.45

## Supplementary Materials

The following are available online: Table S1: First-shell water geometry, Tables S2–S5: Potential parameters. Figures S1 and S2: FEFF input files, Figure S3: Plutonyl EXAFS spectrum form B3LYP simulation obtained by averaging 200 or 500 snapshots, Figure S4: Uranyl EXAFS spectrum from the MP2 optimized geometry vs. experimental ones.
