# Supplementary material for: Combining EXAFS and Computer Simulations to Refine the Structural Description of Actinyls in Water
Source: Molecules. 2020 Nov 11;25(22):5250. doi: 10.3390/molecules25225250 (PMC7697702; doi:10.3390/molecules25225250)
Supplement: Supplementary file 1 [file molecules-25-05250-s001.pdf]

SUPPORTING INFORMATION

Combining EXAFS and Computer Simulations to Refine the Structural Description of Actinyls in Water

Sergio Pérez-Conesa, José M. Martínez, Rafael R. Pappalardo and Enrique Sánchez Marcos

Departamento de Química Física, Universidad de Sevilla,

41012 Sevilla, Spain. e-mail:sanchez@us.es

Table S1: First-shell water geometry in Å and degrees. For Np(V) the first-shell water molecules have the geometry (including the massless particle) of TIP4P. Results are derived from B3LYP, but when indicated NEVPT2.

	First-shell geometry					
	U	Np(VI)	Np(V)	Np(V) (NEVPT2)	Pu(VI)	Am(VI)
$R(O_I - H_I)$	0.97	0.97	0.9572	0.9572	0.97	0.97
$\widehat{H_I O_I H_I}$	106.6	106.6	104.52	104.52	106.6	106.2

Table S2: Effective Merz-Kollmann charges of $[AnO_2{\cdot}(H_2O)_5]^{+/2+}$(aq) using the PCM to model the bulk solvent using as radii 2.65 Å, 1.40 Å and 1.20 Å for An, O and H respectively. For $[NpO_2{\cdot}(H_2O)_5]^{+}$ the first shell water molecules have the same structure and partial charges as the TIP4P model. The electron density used is that of B3LYP (unless when stated NEVPT2)

	Partial Charge (a.u.)					
	U	Np(VI)	Np(V)	Np(V) (NEVPT2)	Pu(VI)	Am(VI)
q_{An}	2.80	2.71		2.52	2.62	2.59
$q_{O_{yl}}$	-0.55	-0.50		-0.76	-0.47	-0.38
q_O	-1.05	-1.04	0.0	0.0	-1.04	-1.06
q_H	0.55	0.55	0.52	0.52	0.55	0.55
q_q	-	-	-	-1.04	-	-

Table S3: Coefficients of the B3LYP (expect when NEVPT2 indicated) IW1 interaction potentials, root mean square error (RMSE) of the fit and shift values. Their units are kcal mol^{-1} Å^{-n}, kcal mol^{-1} and Å respectively.

Coefficients	U	Np(VI)	Np(V)	Np(V) (NEVPT2)	Pu(VI)	Am(VI)
$C_4^{AnO_I}$	-6388.86	781.099	0.0	0.0	-6343.16	-3847.25
$C_6^{AnO_I}$	52787.05	-14881.6	-21667.67	-12254	63274.85	28895.08
$C_8^{AnO_I}$	-131984.94	75576.2	139543.16	72991	-188099.67	-56776.53
$C_{12}^{AnO_I}$	476950.29	-247512	-761189.70	-247770	714067.88	180662.05
δ_{AnO_I}	-	-0.057	-0.069	-	-0.036	-0.069
$C_4^{O_{yl}O_I}$	1696.66	-2989.75	0.0	-702.86	4225.93	1329.48
$C_6^{O_{yl}O_I}$	-8196.99	39404.6	17317.38	17282	-70487.33	-5640.56
$C_8^{O_{yl}O_I}$	18165.85	-140671	-101999.72	-75128	365255.74	318.75
$C_{12}^{O_{yl}O_I}$	-30202.92	637345	756412.76	756410	-2718499.04	235669.93
$\delta_{O_{yl}O_I}$	-	-0.001	-0.030	-	0.030	-0.030
RMSE	0.6	1.4	0.6	0.2	1.6	0.8

Table S4: Coefficients from the fitting of the B3LYP (except when indicated NEVPT2) IMC interaction potentials, $RMSE_{total}$, $RMSE_{partial}$ which is computed for points with energy lower than 15 kcal mol^{-1} with respect to the interaction energy of the minimum and shift values. Their units are kcal mol^{-1} Å^{-n}, kcal mol^{-1} and Å respectively.

Coefficients	U	Np(VI)	Np(V)	Np(V) (NEVPT2)	Pu(VI)	Am(VI)
$C_4^{AnO_{yl}}$	-133370.94	-136859.23	-74453.49	-16380	842.00	-141079.35
$C_6^{AnO_{yl}}$	668178.78	676835.56	403271.32	81524	-28267.67	691027.72
$C_8^{AnO_{yl}}$	-1059978.49	-1067029.16	-695592.42	-125340	74562.59	-1078152.49
$C_{12}^{AnO_{yl}}$	949717.97	953580.31	754700.69	122600	94491.15	938322.18
$\delta_{AnO_{yl}}$	-	0.028	0.046	-	0.053	0.054
$C_4^{O_{yl}O_{yl}}$	-167679.49	-210487.33	-1270195.07	-0.33976E+07	-4881731.10	-305210.33
$C_6^{O_{yl}O_{yl}}$	4369622.37	6034823.14	29013046.5	0.80706E+08	102789274.00	7544789.67
$C_8^{O_{yl}O_{yl}}$	-33177820.27	-45743307.84	-207350887	-0.60584E+09	-680306326.00	-53292782.03
$C_{12}^{O_{yl}O_{yl}}$	605633365.20	779053537.28	3673600800	0.11936E+11	10352292900	840547323.14
$\delta_{O_{yl}O_{yl}}$	-	0.056	0.092	-	0.106	0.108
$RMSE_{total}$	3.4	3.6	3.3	0.3	1.3	2.3
$RMSE_{partial}$	0.4	1.0	1.4	0.001	0.9	0.6

Table S5: Coefficients of the HIW interaction potential from previous work. They are used for all actinyls. $RMSE_{total}$ and $RMSE_{partial}$ which is computed for points with energy lower than 15 kcal mol^{-1} with respect to the interaction energy of the minimum. The units are kcal mol^{-1} Å^{-n} and kcal mol^{-1}.

Coefficients	An(VI,V)	Coefficients	An(VI,V)
$C_4^{UO_W}$	18578.27	$C_4^{O_IO_W}$	-830.40
$C_6^{UO_W}$	-288922.08	$C_6^{O_IO_W}$	6718.50
$C_8^{UO_W}$	1481816.44	$C_8^{O_IO_W}$	-4208.51
$C_{12}^{UO_W}$	-12631787.76	$C_{12}^{O_IO_W}$	-3888.10
$C_4^{UH_W}$	-5094.89	$C_4^{O_IH_W}$	28.22
$C_6^{UH_W}$	65098.47	$C_6^{O_IH_W}$	100.59
$C_8^{UH_W}$	-268274.37	$C_8^{O_IH_W}$	-40.17
$C_{12}^{UH_W}$	1482339.87	$C_{12}^{O_IH_W}$	1.06
$C_4^{O_{yl}O_W}$	-1503.86	$C_4^{H_IO_W}$	-64.73
$C_6^{O_{yl}O_W}$	10373.23	$C_6^{H_IO_W}$	166.44
$C_8^{O_{yl}O_W}$	-15794.55	$C_8^{H_IO_W}$	-57.00
$C_{12}^{O_{yl}O_W}$	12403.99	$C_{12}^{H_IO_W}$	1.25
$C_4^{O_{yl}H_W}$	187.28	$C_4^{H_IH_W}$	4.63
$C_6^{O_{yl}H_W}$	-314.50	$C_6^{H_IH_W}$	-0.29
$C_8^{O_{yl}H_W}$	213.54	$C_8^{H_IH_W}$	0.07
$C_{12}^{O_{yl}H_W}$	-22.69	$C_{12}^{H_IH_W}$	0.00
$RMSE_{total}$	1.9		
$RMSE_{partial}$	1.2		

```
TITLE_U B3LYP Oyl+1a capa

EDGE L3
S02 0.81

CONTROL 1 0 0 0 0 0

PRINT 0 0 0 3 0 0

COREHOLE RPA
EXAFS 18.0

CRITERIA 4.0 2.5
NLEG 4

RPATH 6.0
TDLDA  1
SCF 6.0
EXCHANGE 0 -8.0 0.
POTENTIALS
       0    92   U     3   3
       1    8    Oyl   3   3
       2    8    O     3   3
       3    1    H     2   2

ATOMS
   0.0000000    0.0000000    0.0000000  0  U    0.0000000
  -0.2728680   -0.0778820   -1.7127160  1  Oyl    1.7360641
   0.4805280   -0.1554130    1.6887710  1  Oyl    1.7626707
  -1.0374430    2.1350620    0.5001920  2  O    2.4258957
  -2.4290140   -0.2684420    0.4210760  2  O    2.4798135
  -0.4130360   -2.4453290   -0.1205410  2  O    2.4828940
   2.2708830   -0.9924280   -0.4068550  2  O    2.5114446
   1.7787060    1.9628580   -0.1614320  2  O    2.6538023
  -2.8404220   -0.9764840   -0.0988530  3  H    3.0052105
   2.7044810   -1.3511360    0.3832220  3  H    3.0473997
  -1.7032720    2.5344070   -0.0812680  3  H    3.0546618
  -0.8993450   -2.8170350   -0.8730290  3  H    3.0832916
  -0.8943200    2.7601070    1.2280190  3  H    3.1505602
  -0.4220470   -3.1203030    0.5760410  3  H    3.2009745
   2.6710180    1.7170790   -0.4517060  3  H    3.2072941
   2.6946750   -1.4136170   -1.1709810  3  H    3.2604881
  -3.1531540    0.2434650    0.8141080  3  H    3.2656435
   1.5915230    2.8329280   -0.5472290  3  H    3.2951307
END
```

Figure S1: FEFF input file for the case of the uranyl aquaion: SCF computation.

```
TITLE_U B3LYP Oyl+1a capa

EDGE L3
S02 0.81

CONTROL 0 1 1 1 1 1

PRINT 0 0 0 3 0 0

COREHOLE RPA
EXAFS 18.0

CRITERIA 4.0 2.5
NLEG 4

RPATH 6.0
TDLDA  1
SCF 6.0
EXCHANGE 0 -8.0 0.
POTENTIALS
       0    92   U     3   3
       1    8    Oyl   3   3
       2    8    O     3   3

ATOMS
   0.0000000    0.0000000    0.0000000  0  U    0.0000000
  -0.2728680   -0.0778820   -1.7127160  1  Oyl    1.7360641
   0.4805280   -0.1554130    1.6887710  1  Oyl    1.7626707
  -1.0374430    2.1350620    0.5001920  2  O    2.4258957
  -2.4290140   -0.2684420    0.4210760  2  O    2.4798135
  -0.4130360   -2.4453290   -0.1205410  2  O    2.4828940
   2.2708830   -0.9924280   -0.4068550  2  O    2.5114446
   1.7787060    1.9628580   -0.1614320  2  O    2.6538023
END
```

Figure S2: FEFF input file for the case of the uranyl aquaion: EXAFS signal computation.

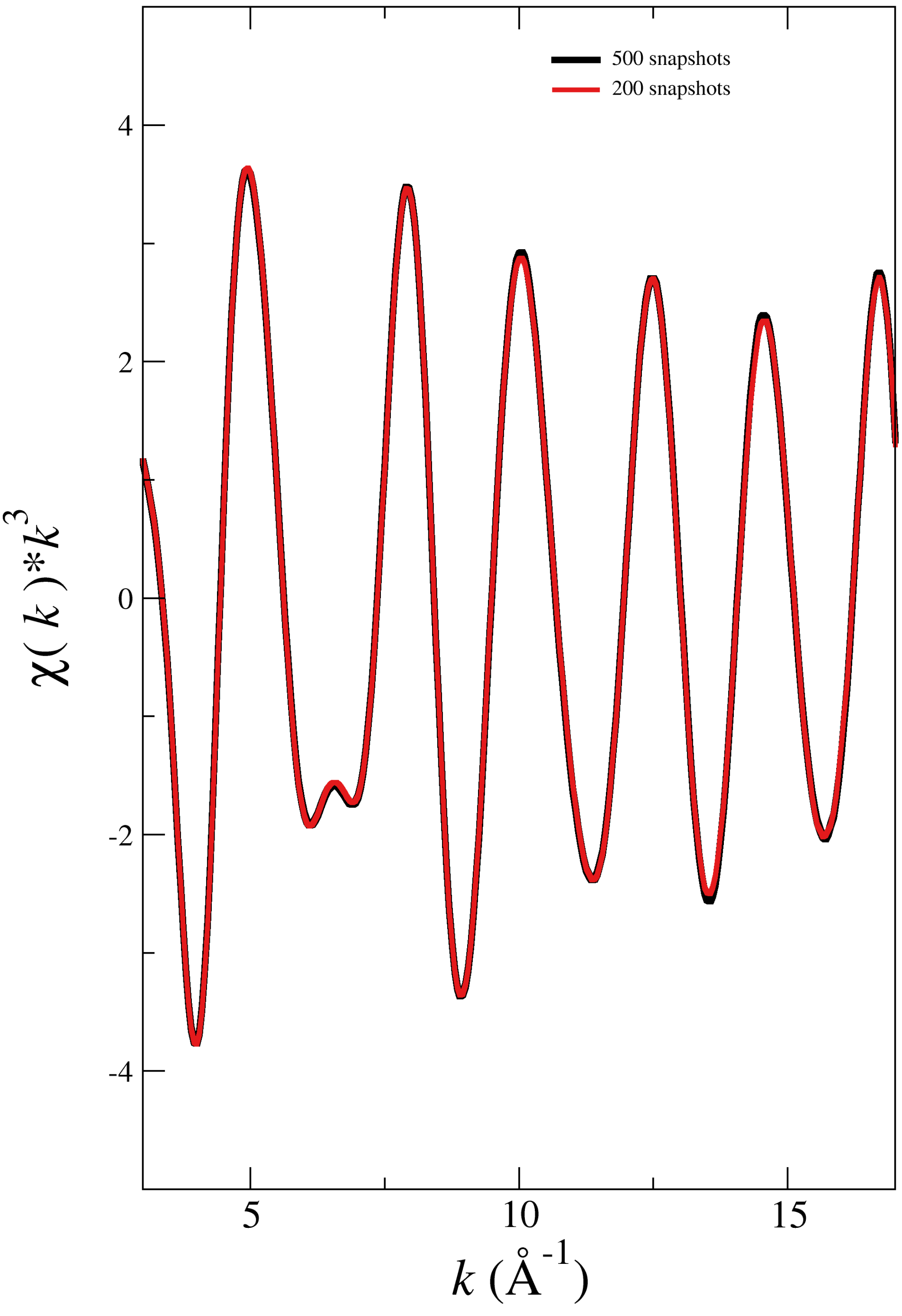

Figure S3: Simulated L_{III}-edge k^3-weighted EXAFS spectrum derived from B3LYP MD simulation of uranyl in water averaging 500 or 200 snapshots.

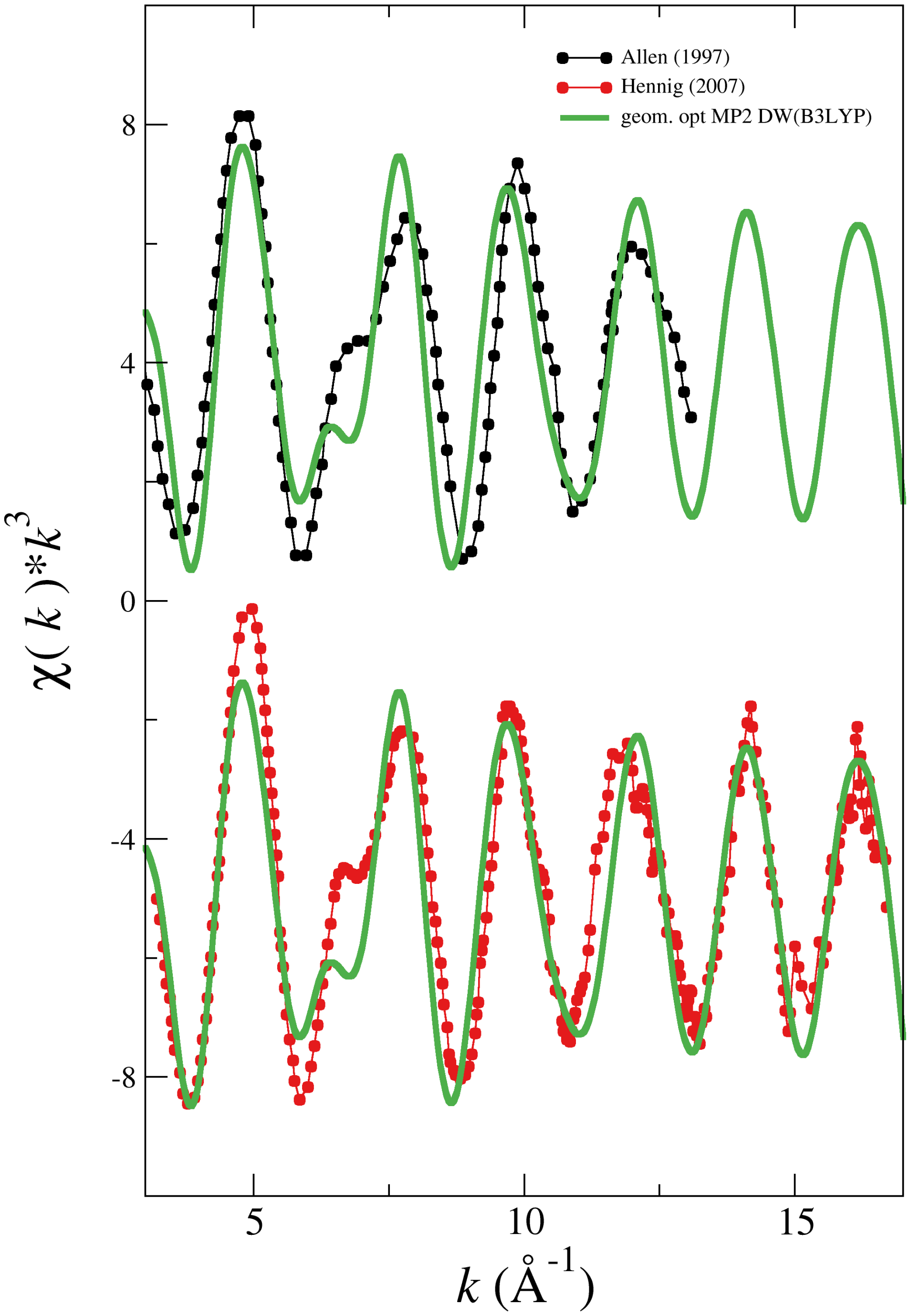

Figure S4: Simulated L_{III}-edge k^3-weighted EXAFS spectrum derived from the MP2 optimized geometry of uranyl in water (green solid line) vs. experimental ones (dots). The experimental EXAFS are taken from Allen et al. *Inorg. Chem.* 36, 4676 (1997), (black dotted line) and Henning et al. *Inorg. Chem.* 46, 5882, (2007) (red dotted line) for uranyl in water.
